# To Kill But Not Be Killed: Controlling the Activity of Mammalian Pore-Forming Proteins

**DOI:** 10.3389/fimmu.2020.601405

**Published:** 2020-11-13

**Authors:** Patrycja A. Krawczyk, Marco Laub, Patrycja Kozik

**Affiliations:** MRC Laboratory of Molecular Biology, Protein and Nucleic Acid Chemistry Division, Cambridge Biomedical Campus, Cambridge, United Kingdom

**Keywords:** immunity, pore-forming proteins, membrane attack complex, perforins, gasdermins, membrane integrity

## Abstract

Pore-forming proteins (PFPs) are present in all domains of life, and play an important role in host-pathogen warfare and in the elimination of cancers. They can be employed to deliver specific effectors across membranes, to disrupt membrane integrity interfering with cell homeostasis, and to lyse membranes either destroying intracellular organelles or entire cells. Considering the destructive potential of PFPs, it is perhaps not surprising that mechanisms controlling their activity are remarkably complex, especially in multicellular organisms. Mammalian PFPs discovered to date include the complement membrane attack complex (MAC), perforins, as well as gasdermins. While the primary function of perforin-1 and gasdermins is to eliminate infected or cancerous host cells, perforin-2 and MAC can target pathogens directly. Yet, all mammalian PFPs are in principle capable of generating pores in membranes of healthy host cells which—if uncontrolled—could have dire, and potentially lethal consequences. In this review, we will highlight the strategies employed to protect the host from destruction by endogenous PFPs, while enabling timely and efficient elimination of target cells.

## Introduction and Overview

The emergence of cell membranes was critical for the evolution of all modern organisms. They provide a physical barrier to separate an organism from its environment and enable compartmentalization of biochemical processes inside cells. In modern multicellular organisms, interfering with membrane integrity is one the most effective strategies employed in immune defense, and membrane disrupting pore-forming proteins (PFPs) have evolved as key effectors in both innate and adaptive immune responses.

PFPs can be found in all kingdoms of life. Bacteria use them to facilitate their entry into cells (e.g., listeriolysin), to aid in the delivery of effector molecules across membranes (e.g., streptolysin O) or as toxic agents (e.g., diphtheria or anthrax toxins) (1). Eukaryotic multicellular organisms, including mammals, use PFPs as either membranolytic pores assembled directly on the surface of invading pathogens or as effectors to selectively eliminate infected or cancerous host cells (2, 3). While bacteria can specifically target eukaryotic membranes through recognition of host-specific molecules, mammals are faced with the more challenging task of eliminating unwanted cells without accidentally damaging surrounding healthy tissues. In fact, mammalian PFPs evolved to show limited target membrane specificity in isolation and therefore depend on other proteins of the immune system to safely guide their activity.

In this review, we discuss how mammalian PFPs are controlled by both the innate and adaptive arms of the immune system. Our goal is to provide a comprehensive overview of the variety of mechanisms, ranging from inducible expression and regulated trafficking to post-translational modifications and proteolytic processing, that collectively ensure tight spatial and temporal regulation of pore formation during immune responses.

### Mammalian PFPs in Innate and Adaptive Immunity

**Figure 1:**
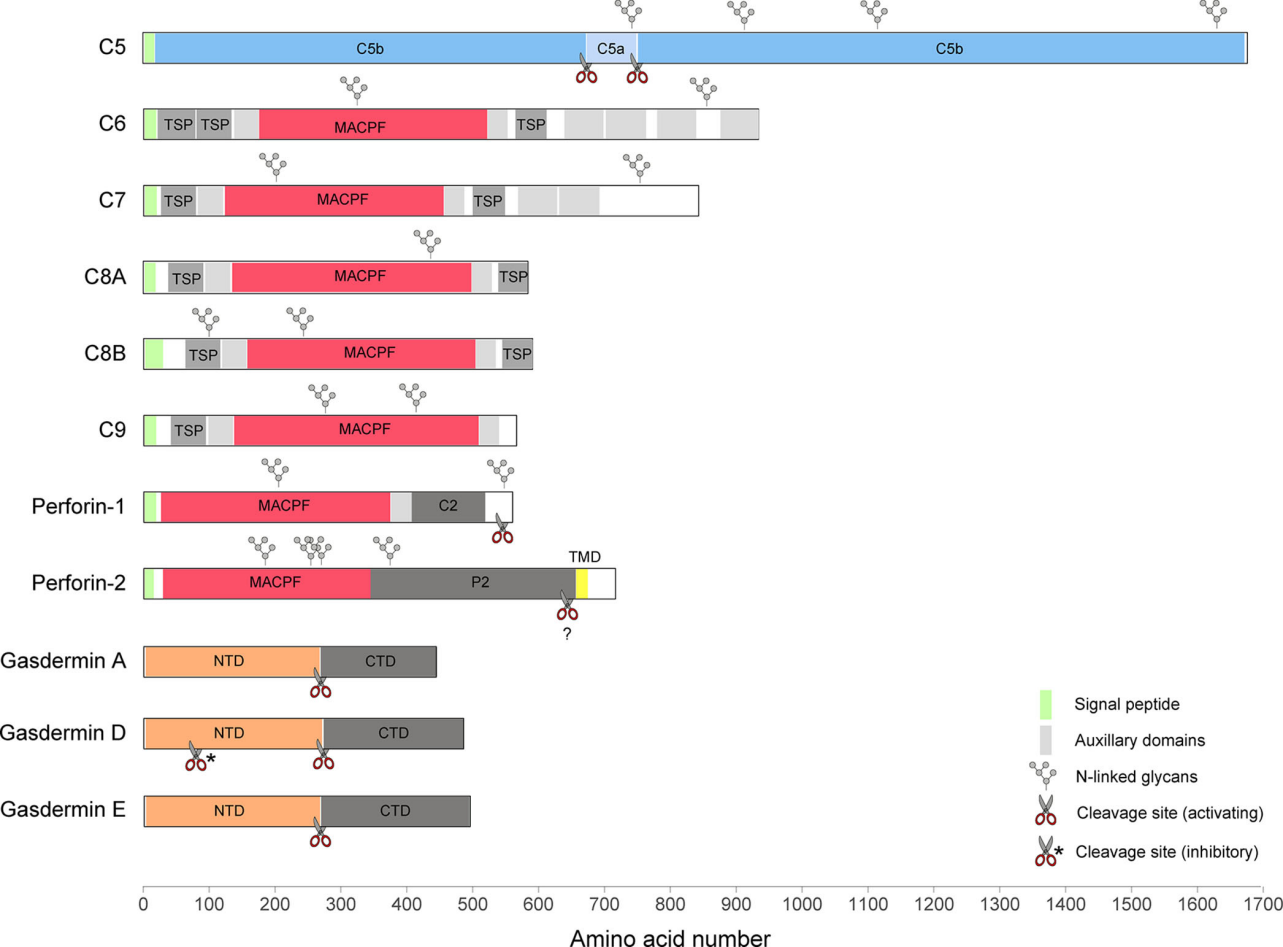
A diagram illustrating the domain structures and selected regulatory features of mammalian PFPs.

Toward the end of the 19th century, George Nuttall and Hans Ernst August Buchner noted that blood contains a heat-sensitive component with killing activity against bacteria (4, 5). The proteins responsible were later named membrane attack complex (MAC) or terminal complement complex. Today we know, that the soluble MAC components C5, C6, C7, C8 (comprising C8α, C8β, and C8γ), and C9 (**Figure 1**) present in the serum assemble to form membranolytic pores on the surface of Gram-negative bacteria, enveloped viruses, parasites, and host cells (6–10). The mediators that initiate the assembly of MAC pores include components of both the innate and adaptive arms of the immune system. Specific receptors of the complement system are able to recognize a wide range of structures including unique pathogen-associated molecular patterns (PAMPs) and microbes opsonized with antibodies (key components of the humoral adaptive immune system) such that MAC can in principle assemble in response to any antigen. Both, the important contribution of MAC to anti-bacterial immunity as well as its potential toxicity are reflected in population genetics. On the one hand, genetic deficiencies in MAC components are associated with susceptibility to neisserial disease including endemic meningococcal infections (11, 12), on the other hand, these deficiencies can also confer a selective advantage (13).

Five of the seven MAC subunits (the exceptions being C5 and C8γ) are evolutionarily related and, together with two perforins, they belong to the membrane attack complex/perforin (MACPF) superfamily of PFPs. Perforin-1 (**Figure 1**) is stored together with pro-apoptotic effectors, granzyme A and B, in specialized secretory granules of natural killer (NK) cells and cytotoxic T cells (CTLs). When released into the immune synapse formed between the cytotoxic lymphocyte and an infected or cancerous cell, it rapidly assembles into homo-oligomeric pores on the target membrane (14). The pores can be lytic at high concentration (15–17), but their primary function is to facilitate entry of granzymes into target cells inducing apoptosis (18). NK cells belong to the innate arm of the immune response and recognize a variety of stress signals presented on the cell surface. In CTLs, key effectors of adaptive immunity, the specificity during immune synapse formation relies on the interaction between the T cell receptor (TCR) and MHC class I complexed with a foreign or a mutated peptide. In line with the fundamental function of perforin-1 in immunity, deleterious variants in the *PRF1* gene in humans lead to an aggressive immunoregulatory disorder called familial hemophagocytic lymphohistiocytosis (FHL, which can be fatal without bone-marrow transplantation) (19, 20), as well as hematological malignancies (21–23). Perforin-1^−/−^ mice exhibit an increased mortality in response to viral infections (24), fail to control tumor growth (25), and are highly prone to the development of spontaneous lymphomas (26).

Perforin-2 (also known as macrophage-expressed gene 1, MPEG1, **Figure 1**) has been discovered only recently (27), but appears to be the most ancient member of the MACPF family. It is encoded by the intronless *MPEG1* gene which is found already in some of the earliest multicellular organisms, such as sponges (28). In mammals, perforin-2 is predominantly expressed in cells of monocytic origin, such as macrophages. During bacterial infection, it is recruited to pathogen-containing vacuoles where it damages membranes of diverse bacterial species limiting their proliferation (29–34). Consistent with its proposed function in antimicrobial defense, four deleterious *MPEG1* variants have recently been identified through whole exome sequencing of patients with pulmonary non-tuberculous mycobacterial infection (35).

The second family of mammalian PFPs, gasdermins (**Figure 1**), has been discovered while searching for molecular mechanisms involved in an inflammatory cell death pathway termed pyroptosis (36, 37). Gasdermins are employed during both innate and adaptive immune responses, for example, in response to inflammasome assembly or following CTL-mediated delivery of granzymes into the cytosol. The best characterized member of this family, gasdermin D (GSDMD), is stored in the cytosol, and when activated, assembles into pores on the plasma membrane. The pores initially facilitate the secretion of cytosolic pro-inflammatory cytokines, such as IL-1β and IL-18, but if they persist unrepaired, the cell undergoes pyroptosis (38–40). In neutrophils, GSDMD pores contribute to the generation of neutrophil extracellular traps (NETs), secreted chromatin structures which capture extracellular pathogens (41). *In vitro*, GSDMD has also been shown to target cytosolic bacteria directly (38, 42).

In total there are six gasdermin paralogues in humans (*GSDMA*, *GSDMB*, *GSDMC*, *GSDMD*, *GSDME*/*DFNA5*, and *PJVK*/*DFNB59*) and 10 in mice (*Gsdma1*-3, *Gsdmc1*-4, *Gsdmd*, *Gsdme*, and *Pjvk*). Notably, gasdermin orthologues are also found in lower vertebrates, such as zebrafish (43), and more distant homologues are present in fungi (44). Mutations in gasdermin genes have been associated with a variety of disease phenotypes including skin and developmental defects (*GSDMA3*), susceptibility to asthma (*GSDMA3* and GSDMB) (45–47), and autosomal dominant and recessive hearing loss (*GSDME* and *DFNB59*) (48, 49). The precise functions, mechanisms of activation, and physiological relevance for the majority of the gasdermins remain to be uncovered.

### General Mechanism of Pore Formation

Mammalian PFPs are synthesized and stored in an inactive conformation as monomeric, usually soluble proteins. Structural studies revealed that the pore-forming fold of the MACPF proteins is highly similar to the pore-forming domain of bacterial cholesterol-dependent cytolysins (CDC) (50–52). In contrast, the pore-forming N-terminal domain of gasdermins is structurally distinct from the MACPF/CDC fold and is thought to have evolved independently (53, 54). Nevertheless, the MACPF/CDC and gasdermin family members follow a broadly similar mechanism of pore formation which can be roughly divided into three stages: membrane binding, oligomerization, and membrane insertion (55, 56). Membrane insertion is accompanied by dramatic structural rearrangements that include refolding of α-helical regions into transmembrane β-hairpins, termed TMH1 and TMH2 in MACPF/CDC proteins, and HP1 and HP2 in gasdermins. In the resulting β-barrel pores, each monomer typically contributes one four-stranded lipid-embedded β-sheet.

### Challenges in Storing and Targeting of Mammalian PFPs

Despite considerable similarity in the pore assembly process, the mechanisms involved in selecting target membranes differ strikingly between bacterial CDCs and mammalian MACPF proteins and gasdermins. For bacterial PFPs, the transition between soluble monomer and membrane pore is initiated by binding of the PFP to proteins, sugars, or lipids unique to the host. Thus, CDCs form pores preferentially on membranes with 25–35% cholesterol content, a lipid present only in eukaryotic cells (57). As mammalian PFPs themselves show limited target membrane selectivity, additional mechanisms need to be in place to enable spatiotemporal control of pore formation and to limit damage to both PFP-producing cells and surrounding tissues.

## Transcriptional Control of PFP Expression

**Figure 2:**
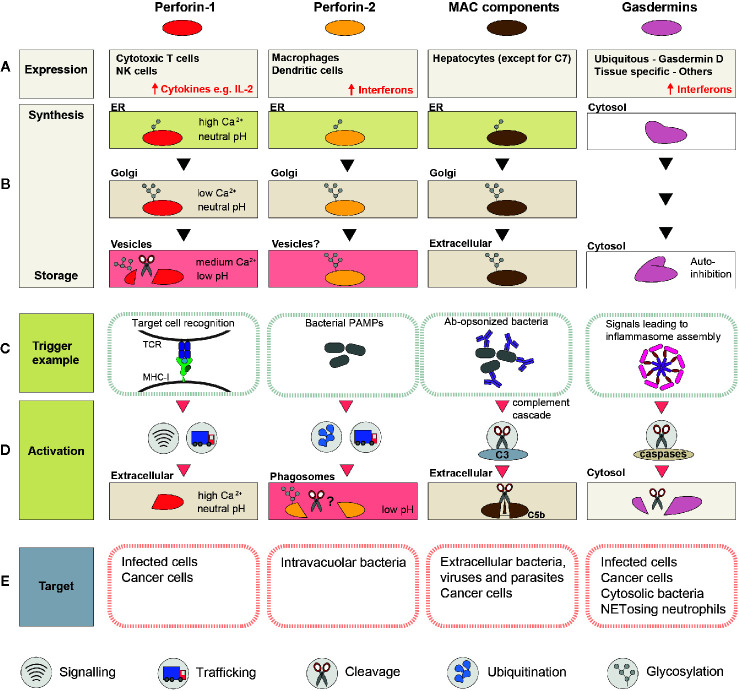
A schematic summary of the mechanisms involved in regulation and activation of perforin-1, perforin-2, MAC, and gasdermins. **(A)** Cell types or tissues in which the PFP is constitutively expressed (inducible expression is highlighted with ↑). **(B)** Processing and trafficking steps involved in synthesis of the “stored” form of the PFP. The last row corresponds to the storage compartment. **(C)** Immune system components which initiate pore formation, and ligands they recognize. **(D)** A schematic representation of the events that precede pore assembly. **(E)** Targeted membranes.

While some PFPs can be safely expressed in the majority of cells (e.g., gasdermins), others (e.g., perforins) can be toxic soon after translation and are only produced by specialized cells of the immune system (**Figure 2A**).

### MAC Components Are Produced in the Liver

The majority of the MAC components, similar to other serum proteins, are produced by hepatocytes in the liver (58). The exception is C7, which is expressed primarily extrahepatically as shown by the fact that, in liver transplant patients, as little as 10% of plasma C7 originated from the donor cells (59), compared to nearly 100% for C6 and C9 (60, 61). It has been proposed that local synthesis of C7 might be important for modulation of MAC activity (62), but this hypothesis has not yet been verified with tissue- or cell type-specific C7 knockouts. Intriguingly, liver-derived C7 is actually not produced by hepatocytes, but by endothelial and stellate cells (63, 64), raising the question whether co-expression of C7 with the remaining MAC components might be toxic for the cells.

### Gasdermins Can Be Expressed Ubiquitously or in a Tissue-Specific Manner

Members of the gasdermin family employ a wide range of different transcription factors to regulate their constitutive and inducible expression, and as a result, they all display different expression patterns [summarized in a recent review by Broz et al. (65)]. GSDMD and GMDMB are the most abundant and ubiquitous family members, while expression of the remaining gasdermins is more restricted. GSDMA, for example, is predominantly expressed in the skin, while GSDME is mainly present in the blood, spinal cord and uterus (58). Expression of gasdermins can be further regulated during a variety of pathological conditions, such as infection (66), cancer (67, 68), or in response to DNA damage (69). For example, GSDMD expression is strongly upregulated during bacterial, viral, and parasitic infections by interferon-γ-dependent signaling pathways (66).

### Perforin-1—an Effector Restricted to Killer Lymphocytes

In contrast to MAC components and gasdermins, perforins are primarily expressed in immune cells. *PRF1* promoter activity is restricted to the T and NK cell lineages during development (70). In T cells, *PRF1* expression is induced upon maturation of naïve T cells into CTLs during an active immune response [reviewed in (71)]. T cell activation requires three distinct signals delivered by dendritic cells: signal 1—MHC class I-bound peptides, identical to those present in target cells (recognized by TCR); signal 2—cell surface molecules that act as stimulatory or inhibitory co-receptors, and signal 3—chemokines and cytokines that modulate T cell proliferation and differentiation. These requirements ensure that only T cells with relevant TCRs are activated, expanded, and upregulate perforin-1 (72). Mechanistically, regulation of perforin-1 transcription is a complex process controlled by a 150 kb territory surrounding the *PRF1* gene (70) and several transcription factors (Sp-1, AP-1, MEF, MITF, T-bet, and EOMES) [reviewed in (73, 74)].

NK cells upregulate *Prf1* expression early during their development (75, 76). Yet, resting NK cells isolated from mice bred under pathogen-free conditions are only minimally cytotoxic and they contain only a small amount of perforin-1 protein (77). Instead, they store large quantities of *Prf1* mRNA, which is only translated upon NK cell activation (e.g., by cytokines IL-15 or IL-2). How mRNA translation is regulated in NK cells is not fully understood. The lymphocyte specific miRNA, miR-150, has been shown to target *Prf1* mRNA and downregulate its translation in resting NK cells (78), but additional mechanisms involving cytoplasmic mRNA-binding proteins and mRNA modifications are likely to be involved (79, 80).

### MPEG1 Is Expressed Predominantly in Antigen-Presenting Cells

*MPEG1* is constitutively expressed in macrophages, dendritic cells, monocytes, and granulocytes (81), but interferon-inducible expression has been observed in other cells types, e.g., fibroblasts and neurons (32, 82). Thus, *MPEG1* is strongly upregulated during bacterial, viral, and parasitic infections. For example, in the lungs of mice infected with influenza virus, it is among the top 30 genes with highest expression (66). Interestingly, pathogens that disrupt interferon signaling during infection have been reported to escape perforin-2-mediated killing. This has been demonstrated for *Chlamydia trachomatis*, which prevents interferon-dependent translocation of the STAT1 transcription factor into the nucleus (83). While ectopic expression of *MPEG1* in HeLa cells suppresses chlamydial growth, *MPEG1* is upregulated only in cells exposed to heat-killed *Chlamydiae*, but not live bacteria (29).

## Maturation and Storage

Newly synthesized PFPs are rarely immediately activated. Instead, they are stored as inactive monomers that can be rapidly deployed in response to the appropriate signals (**Figure 2B**). These nascent PFP are unable to bind membranes as monomers, are synthesized as inactive immature propeptides, and/or are stored in a compartment where the ionic environment is incompatible with pore formation.

### MAC Components Are Abundant in the Plasma

All seven MAC subunits are secreted into the plasma and circulate the body through the blood vessels. C8 isolated from plasma is a stable heterotrimer of C8α, C8β, and C8γ (with C8α covalently bound to C8γ *via* a disulfide bridge) (84), but the remaining subunits are monomeric. *In vitro*, C9 can be forced to form homooligomeric pores (85, 86) but the poly-C9 complex is unable to insert into cell membranes (85), and under physiological conditions, the TMH1 domain of C9 precludes polymerization in the absence of other MAC components (87). The secreted MAC components do not bind membranes efficiently on their own and as a consequence they are non-lytic at steady-state. C5 is the only MAC subunit which is proteolytically processed prior to pore formation, and this cleavage event is what initiates a cascade of conformational changes that drives MAC assembly.

### Gasdermins Are Stored as Immature Propeptides in the Cytosol

In contrast to MAC components, gasdermins are synthesized and stored as immature cytosolic propeptides, which adopt an auto-inhibited state immediately after folding (38, 54, 88). Gasdermins contain an N-terminal pore-forming domain (NTD) connected to an inhibitory C-terminal domain (CTD) by a flexible linker (with the exception of PJVK/DFNB59 which has a truncated C-terminus). Proteolytic cleavage at the linker region destabilizes the NTD-CTD intramolecular interaction releasing the NTD to assemble into pores (36, 37). Indeed, ectopic expression of the NTD alone is sufficient to trigger pyroptotic cell death (42). It has been concluded that the CTD masks the lipid-binding motif of the NTD preventing membrane association of full-length gasdermins (54), but it does not sterically hinder pore formation. Mutations, including known disease variants, that weaken the NTD-CTD interdomain interactions are sufficient to expose the lipid binding motif and trigger constitutive gasdermin activity without cleavage (38) raising a possibility of alternative activation mechanisms.

### Fully Processed Perforin-1 Is Stored in Cytotoxic Granules

Perforin-1 is also initially synthesized as an inactive propeptide but, in contrast to gasdermins, it is stored in a mature form. Besides the MACPF domain, immature perforin-1 consists of two additional domains: an epidermal growth factor (EGF) domain, and a C-terminal, Ca^2+^-binding domain (C2) required for association with the membrane (89, 90). Perforin-1 maturation involves cleavage of a short (~2 kDa) fragment of the C-terminal domain which contains a bulky Asn549-glycan (91). The Asn549-glycan inhibits pore formation by steric hindrance interfering with oligomerization (92). Indeed, full length (uncleaved) perforin-1 purified from human NK KHYG1 cells does not form pores (92), while the full-length Asn549-glycosylation deficient mutant is lytic when purified and toxic when overexpressed (93). Intriguingly, full-length perforin-1 purified from insect cells is fully functional (52, 93), suggesting that the size of the glycan moiety is critical for inhibition of perforin-1 activity [N-glycoproteins produced in standard insect cell expression systems acquire simple side-chains instead of complex N-glycans found in mammalian proteins (94)]. Similarly, the unbranched N-glycans acquired initially in the ER offer only partial protection from unwanted lysis, and N-glycosylated perforin-1 is still toxic when ER exit is slow (e.g., in BFA-treated cells or upon fusion of an ER retention signal to perforin-1 C-terminus) (93).

Following further branching of glycans in the Golgi, perforin-1 is transported into lysosome-related organelles, called cytotoxic granules. Once in the granules, lysosomal proteases cleave the C-terminus, along with the inhibitory Asn549-glycan, to prime perforin-1 for activation upon secretion. *In vitro*, perforin-1 can be cleaved by cathepsins L or B (95, 96), but *Ctsl^−/−^* or *Ctsb^−/−^* mice do not show a defect in perforin-1-mediated killing (96, 97). In contrast, mice lacking asparaginyl endopeptidase (AEP) show degranulation defects, but the potential contribution of AEP to perforin-1 cleavage has not been addressed directly (98). Consistent with the potential involvement of multiple (or redundant) proteases, the cleavage site itself does not appear to be precise and mass spectrometry analysis of perforin-1 immunoprecipitated from KHYG1 cells identified fragments with multiple C-termini (92). In line with this observation, site-directed mutagenesis of the C-terminal region did not reveal any critical positions and cleavage occurred even when every residue from Gln540 through to Gly548 was replaced with a serine. Thus, perforin-1 proteolytic processing may be mediated by non-specific lysosomal proteases and the susceptibility of the region to cleavage is likely to be due to its disordered character.

Regardless of the exact nature of the protease, the cleavage takes place only after perforin-1 reaches a low pH compartment (91). This is critical, as acidic pH (~5.5 in the granules) prevents the fully processed perforin-1 from pore-formation (89). At pH < 6.2 the key acidic residues involved in ionic interactions between monomers are protonated preventing oligomerization (14). Furthermore, protonation of Ca^2+^-binding Asp residues is expected to reduce Ca^2+^ binding to the C2 domain, which is critical for the association of perforin-1 with the target membrane (89, 90, 99). Ca^2+^ binding might be additionally prevented by granule-resident calreticulin, which sequesters free Ca^2+^ ions to further inhibit perforin-1 activity (100). Thus, cleavage only occurs when perforin-1 reaches a compartment with an environment incompatible with pore formation, providing an important safeguarding mechanism.

### Where Is Perforin-2 Stored at Steady State?

Perforin-2 is a type I transmembrane protein comprising the MACPF domain, a unique membrane-binding P2 domain, a transmembrane domain, and a short cytosolic tail. The protective mechanisms involved in its biosynthesis and storage are not well characterized. In membrane-anchored perforin-2, the pore-forming TMH regions would face away from the membrane after unfurling (101, 102), suggesting that the transmembrane domain might be important for preventing perforin-2 assembly on host cell membranes. This unusual topological feature might therefore offer an elegant safeguard against accidental autolysis during biosynthesis and storage.

Overexpressed GFP- or RFP-tagged perforin-2 shows diffuse staining, which could correspond to small post-Golgi vesicles (29, 32). Hence, it appears that similar to perforin-1, perforin-2 might also be sorted into a specialized storage compartment. However, in contrast to perforin-1, the ectodomain of perforin-2 forms pores at low pH (101, 102), suggesting that the hypothetical storage compartment would have to have elevated rather than low luminal pH.

Intriguingly, overexpression of perforin-2 in HEK-293Ts is toxic (28, 103), yet in professional phagocytes it is one of the most abundant proteins. For example, in mouse dendritic cells, perforin-2 is ranked as the top 55^th^ protein by abundance (104) with an estimated 3x10^6^ copies safely stored within each cell (105). It appears likely, therefore, that antigen-presenting cells (APCs) employ specialized mechanisms to protect their intracellular membranes from perforin-2 mediated damage, but this has not been addressed to date. Considering that in non-immune cells, perforin-2 is expressed during infection or upon stimulation with interferons (30, 32), it is possible that interferons also facilitate expression of additional factors that control perforin-2 activity.

## Initiation of Pore Formation

Despite broadly similar mechanisms of pore formation, the signals that trigger PFP activation and the events that precede pore formation are strikingly different between MAC, perforins, and gasdermins (**Figures 2C, D**).

### MAC: Cleavage of C5 Triggers a Cascade of Conformational Changes

MAC assembly can be initiated by multiple enzymatic chain reactions known as the classical, lectin and alternative pathways [reviewed in (106)]. All three pathways converge on the formation of a C5 convertase, which catalyzes the cleavage of C5, the key event in initiation of MAC assembly. The activation of the classical pathway starts when the C1q component of the complement system binds to antigen-antibody complexes, e.g., IgM- or IgG-opsonized bacteria. The lectin pathway is initiated following the recognition of pathogen-specific carbohydrates on the bacterial surface (e.g., by mannose-binding lectins, collectins, or ficolins). Ligand-bound C1q or lectin receptors initiate distinct proteolytic cascades, but both lead to cleavage of C4 and C2. The resulting cleavage products, C4b and C2a, assemble into the C3 convertase, which then cleaves C3 to generate C3b. The C4bC2aC3b complex forms the classical C5 convertase. The alternative pathway starts with spontaneous C3 hydrolysis or by deposition of C3b directly on bacterial surface during ongoing complement activation. These events, in the presence of factors B and D, lead to formation of the alternative C5 convertase, C3bBbC3b.

The cleaved C5 initiates a cascade of conformational changes that lead to MAC assembly and unfurling of the TMH domains. Cleavage generates a small C5a fragment (74-77 residues in length) and a large 170 kDa C5b fragment formed by two peptide chains, β (residues 19–673) and α (residues 752–1676), linked by a disulfide bond. C5b is very labile and it decays (aggregates) within 2 min unless stabilized by binding of C6 (107). The C5bC6 associates weakly with the membrane but remains soluble as the TMHs of C6 are not yet deployed in the dimer (108). Membrane binding interfaces may instead be provided by auxiliary domains such as C6 thrombospondin-like domain which has amphipathic properties at its base (109). The C5bC6 complex recruits C7 driving a cascade of conformational changes within auxiliary domains of C6 and C7 that ultimately trigger unfurling of TMHs in both C6 and C7 and membrane anchoring (110–113). The C6 and C7 β-hairpins, however, do not fully penetrate the membrane. Instead, recruitment and irreversible binding of the C8 trimer and unfurling of the four additional β-hairpins (in C8α/C8β) leads to formation of a stable C5bC6C7C8 complex that can no longer be removed from the membrane by washing (114, 115). The C5b-C8 complex initiates polymerization and membrane insertion of up to 18 copies of C9, recruited directly from solution to the growing pore (111). Notably, MAC pores are the only known PFPs that form hetero-oligomeric, asymmetric pores.

### Gasdermins Are Cleaved to Release a Pore-Forming Domain

Gasdermin pores are formed following a regulated cleavage event of the immature propeptide. This cleavage is primarily executed by caspases, which themselves are stored as inactive propeptides that require proteolytic processing for activation.

GSDMD is primarily cleaved by caspases-1 and -4/5/11 (36, 37). Caspase-1 is activated by so-called canonical inflammasomes, a group of large multiprotein complexes composed of distinct pattern recognition receptors (LRP1, NLRP3, NLRC4, AIM2, and pyrin). Canonical inflammasomes assemble in response to a wide range of PAMPs and damage-associated molecular patterns (DAMPs), including bacterial flagellin, cytosolic dsDNA, ROS, ionic imbalances, and many others [reviewed in (116)]. By contrast, caspases-4 and -5 (and caspase-11 in mice) are activated within non-canonical inflammasomes by directly binding to bacterial lipopolysaccharides (LPS) (117). Activated, proteolytically processed caspases-1/4/5/11 bind to the GSDMD C-terminal domain executing cleavage within the GSDMD linker region at position Asp276, in the Phe-Leu-Thr-Asp (humans) or Leu-Leu-Ser-Asp (mice) tetrapeptide (118, 119). In *Yersinia*-infected cells, a caspase-8-dependent processing of GSDMD has also been reported (120, 121). However, considering the low efficiency with which caspase-8 cleaves GSDMD *in vitro*, it is unclear whether the observed effect was specifically due to cleavage of GSDMD or other co-factors (121, 122). Other proteases implicated in GSDMD activation include neutrophil elastase (released from cytoplasmic granules) (123, 124) and cathepsin G (released from lysosomes) (125), but the physiological relevance of these pathways remains to be addressed.

Non-inflammatory signals might also regulate the activity of gasdermins. For example, GSDME can be cleaved and activated by caspase-3, an executioner of canonical apoptosis. While this cleavage results in conversion of apoptotic cell death into pyroptosis (39, 40), intriguingly, caspase-3-mediated cleavage of GSDMD at position Asp87 inhibits pore formation, and as a result inhibits pyroptosis (40, 122, 126). Furthermore, two recent studies suggested that granzymes B and A, delivered by CTLs/NKs through perforin-1 pores, can cleave gasdermins E and B, respectively, and promote pyroptotic rather than apoptotic death of cancer cells (127–129). Hence, unexpectedly, gasdermin-mediated pyroptosis might also contribute to CTL-mediated killing during adaptive immune responses. These findings reveal an intriguing cooperation between mammalian PFPs and further strengthen the notion of plasticity between cell death pathways.

### Perforin-1 Is Released From Cytotoxic Granules Into a Neutral pH Environment

The key event that triggers the assembly of perforin-1 pores involves the formation of an immune synapse between a CTL/NK cell and the target cell, followed by fusion of the lytic granules with the plasma membrane and secretion of their contents into the synaptic space.

For CTLs, secretion of the cytotoxic granules is triggered by the association of antigen-specific TCR with MHC I loaded with a foreign or a mutated peptide. Exocytosis of NK granules is controlled by integration of signals delivered from activating and inhibitory cell surface receptors [reviewed in (130)]. The best characterized inhibitory receptors, killer immunoglobulin-like receptors (KIRs) in humans (Ly49 in mice), also bind MHC I and are maximally engaged at the MHC I density found on healthy host cells (131). This, therefore, provides a mechanism to specifically target cells that escape CTL killing by downregulating MHC I—an evasion mechanisms employed by both viruses (132) and cancers [reviewed in (133)]. The NK activating receptors recognize either host proteins upregulated in response to cellular stress (134, 135) or viral proteins expressed on the cell (e.g., viral hemagglutinins bound by receptors NKp46 and NKp44).

The precision in killing is ensured by polarized secretion of the cytotoxic granules toward the immune synapse [reviewed in (136–139)]. The polarization depends on the phospholipase C-γ and Ca^2+^-dependent signaling of the TCR or NK activating receptors and is followed by dynein-dependent movement and docking of the microtubule organizing center at the synapse. Subsequent microtubule-dependent transport and exocytosis of the granules release perforin-1 from its inhibitory storage compartment into an environment highly favorable for pore formation. The high extracellular Ca^2+^ concentration (~1–1.3 mM) stabilizes the perforin-1 C2 domain and induces a conformational change that permits four key hydrophobic residues to anchor perforin-1 to the plasma membrane of the target cell (99). The neutral pH further facilitates ionic intermolecular interactions of perforin-1 monomers driving their oligomerization into ring- and arc-shaped pores (14, 140, 141).

### Perforin-2 Pores Are Assembled at Low pH

Perforin-2 activity has been observed only at low pH *in vitro* and is likely controlled by regulated trafficking to an acidic compartment. Indeed, RFP-tagged perforin-2 redistributes to bacteria-containing phagosomes during infection with *Escherichia* *coli* or *Salmonella* *typhimurium* (32). In cells infected with enteropathogenic *E. coli* strain E2348/69 or treated with LPS, the C-terminal cytosolic tail of perforin-2 is monoubiquitinated by a cullin-RING E3 ubiquitin ligase (CRL) complex and mutation of the lysines in the cytosolic tails of perforin-2 prevents its recruitment to phagosomes and bactericidal activity (31). Yet, the signaling pathways that promote ubiquitination, the mechanisms involved in trafficking of ubiquitinated perforin-2 or whether ubiquitination is indeed required for perforin-2 recruitment to pathogen-containing vacuoles remain to be carefully addressed.

Several lines of evidence suggest that the activation of perforin-2 in acidic compartments might involve cleavage of its ectodomain from the transmembrane domain. Firstly, the ectodomain alone assembles into pre-pores and pores on liposomes *in vitro* (101, 102). Secondly, in HEK-293 cells perforin-2 was able to form ring-like structures only following trypsin treatment (32). Finally, perforin-2 that was present on bacteria isolated from MEFs expressing perforin-2-GFP was recognized by antibodies specific to MACPF and P2 domains but not to the cytosolic tail (32). Nevertheless, neither the cleavage site nor the relevant proteases have been identified to date and future work will need to address whether ectodomain release is indeed physiologically relevant.

Intriguingly, perforin-2 does not restrict bacterial growth without pre-stimulation of cells with IFN or LPS (32). It is not known, however, whether pre-stimulation is required for processing/trafficking of perforin-2 itself, whether it stimulates expression of co-factors required to trigger pore formation, or whether the pore forming ability of perforin-2 is insufficient to restrict bacterial growth in the absence of additional interferon-stimulated genes that facilitate killing of pathogens.

## Safety Mechanisms for Protection of Bystander Membranes

After the appropriate trigger signals have been received, the newly unleashed lytic activity of PFPs requires continuous control as unrestrained pore formation would not only be highly damaging to bystander membranes but would also reduce the availability of monomers for a productive lytic response at the target membrane (**Figure 2E**).

### Lipid-Binding Selectivity of Gasdermins Prevents Bystander Cell Lysis

Gasdermins preferentially bind to negatively charged lipid species [cardiolipin, phosphatidylinositol phosphates (PIPs), phosphatidic acid (PA), and phosphatidylserine (PS)] which are found on the inner leaflet of the plasma membrane but are absent from its extracellular leaflet (38, 39, 42). This lipid-binding preference therefore appears to be sufficient to protect bystander cells from activated gasdermins released during pyroptosis (142). Given that the cytosolic leaflets of endosomes and phagosomes contain the same lipid species as the inner leaflet of the plasma membrane, it is likely that gasdermins pores are not restricted to the plasma membrane. Whether intracellular membranes [other than the mitochondrial membrane (143)] are indeed disrupted by gasdermins and, if not, how they are protected remains to be investigated.

### Inactivation of MAC Assembly

The soluble C5bC6 complex can in principle diffuse away from the target membrane and initiate pore assembly on bystander cells. MAC formation, however, can be inhibited at multiple stages during pore formation, even after proteolysis of C5. The key factors that disarm MAC pores include CD59, clusterin, and vitronectin.

CD59 is a small GPI-anchored glycoprotein widely expressed on the surface of mammalian cells (58). CD59 inhibits MAC formation by binding to C8 in the C5b-8 complex thus preventing C9 incorporation, as well as by binding to C9 in the preformed C5b-9 complex suppressing further polymerization (144). Specifically, CD59 interacts with C8α and C9b in regions exposed during MAC formation (114, 145). The protective role of CD59 is most evident when its levels are pathologically low. Deficiencies in CD59 or in proteins required for biosynthesis of its GPI anchor result in inflammatory neuropathy, recurrent strokes, and chronic hemolysis (146–148).

In contrast to CD59, clusterin and vitronectin are soluble glycoproteins found in plasma (149). Clusterin can inhibit the lytic activity of C5b-7, C5b-8, and C5b-9 subcomplexes by interacting with C7, C8β, and C9 through binding sites exposed during pore formation (150). Vitronectin, also known as S-protein, has been reported to inhibit MAC insertion at two stages: either by binding to the nascent C5b-7, rendering the complex soluble (151) or by inhibiting polymerization of the C9 subunits (152). Interestingly, some Gram-negative bacteria including *Moraxella catarrhalis*, *Haemophilus influenzae*, and *Neisseria gonorrhoeae* recruit vitronectin to prevent MAC deposition on their surface and escape MAC-mediated killing [reviewed in (153)].

### How Are Cytotoxic Lymphocytes Protected From Perforin-1 at the Immune Synapse?

Both CTL and NK cells can sequentially kill several target cells suggesting that the killing cell itself does not undergo a bystander death (154–157). Indeed, several studies have demonstrated that various T cell lines as well as primary T cells are more resistant to killing by other CTLs compared to, for example, cancer cell lines (158–160). A similar increased resistance was demonstrated to granule extracts and purified perforin-1 alone (158, 161, 162). Nevertheless, lymphocytes are not invulnerable to CTLs, especially when the attack is directed against them (160, 163, 164). This so-called fratricide (i.e., killing of CTLs by CTLs) might not only be crucial to eliminate CTLs that have been infected or accumulated mutations, but could also help to dampen an excessive immune response (165). Importantly, when an immune synapse between two CTLs is formed, only one cell gets polarized to inflict cell death and the killer always survives (166). Moreover, while a CTL engaged with the target cell avoids destruction by their own lytic mediators, it is not refractory to bystander lysis when induced by neighboring CTLs (167). This apparent paradox suggests that cytotoxic lymphocytes acquire additional resistance to perforin-1 primarily within the immune synapse following degranulation.

Several models were proposed to explain why the degranulating lymphocyte might be resistant to perforin-1, but none has been widely accepted to date. Earlier studies suggested that other proteins contained within the granules might have protective functions during degranulation. For example, Balaji et al. (95) observed that CTLs are more prone to death in the presence of membrane impermeable cathepsin B inhibitors and proposed that secreted cathepsin B cleaves perforin-1 on the surface of degranulating CTLs to protect them. More recent work, however, revealed that CTLs of cathepsin B-null mice survive their encounter with target cells normally (97). Jiang et al. (168) suggested in turn that glycosylation and sialylation of membrane protein(s) on the CTL surface might provide negative charges that repel perforin-1 and in later work by Cohnen et al. (169), LAMP1/CD107a was implicated as a key O-glycosylated and sialylated protein involved. In line with this model, LAMP1 deficient NK cells were more susceptible to apoptosis after an encounter with the target and overexpression of truncated LAMP1 (targeted directly to the cell surface) reduced apoptosis caused by cytotoxic granules. A separate study, however, did not report a reduction in perforin-1 binding to the surface of primary mouse T cells that overexpress LAMP1 (162). Considering the putative role of LAMP1 in trafficking perforin-1 toward cytotoxic granules (170), the exact contribution of LAMP1 to preventing perforin-1 mediated damage might be difficult to decipher.

Alternatively, perforin-1 resistance could be conferred by local changes in lipid composition that follow degranulation. This model is supported by the observations that perforin-1 preferentially forms pores on phosphatidylcholine-rich, disordered lipid phases, avoiding sphingomyelin/cholesterol-rich ordered domains abundant within the immune synapse (162, 171–173). Furthermore, degranulation is associated with a transient increase of surface exposed PS which is also believed to provide a membrane composition unfavorable for pore assembly (174, 175). On the one hand, the presence of PS could simply interfere with perforin-1 membrane binding (42). On the other hand, PS might act as a negatively charged sink that binds perforin-1 in a conformation incompatible with pore assembly (162).

Finally, unidirectional killing might be facilitated by mechanopotentiation, the process of increasing membrane tension on the target cell *via* the exertion of synaptic forces (176). The forces at the immune synapse are generated by the concerted action of cytosolic proteins that regulate actin dynamics, myosin II, and integrins (177–179). A resulting increase in membrane tension on the target cell was proposed to lower the necessary concentration of perforin-1 required for pore assembly (176). This discovery implies an additional function of the immune synapse in protecting from perforin-1-mediated damage: not only does it protect bystander cells by limiting perforin-1 diffusion, but also the cytotoxic cells themselves, by lowering the effective concentration of perforin-1 required for pore assembly on the target membrane.

### (How) Are Phagosomal Membranes Protected From Perforin-2 Activity?

Little is known about the mechanisms involved in the protection of host cells from perforin-2 pores formed in phagosomes. *In vitro*, perforin-2 displays preference for negatively charged lipids including PS, PIPs as well as cardiolipin, which is found in the membranes of most bacteria (101, 102). However, considering that overexpressed perforin-2 can be toxic (103) and that perforin-2 pores have been observed also on mammalian membranes (32), it is unclear whether in infected cells the pores are solely formed on the pathogen surface.

### Pore Insertion and Membrane Repair Pathways

Even a small injury to the plasma membrane can lead to local spikes in cytosolic Ca^2+^ and trigger membrane repair pathways in the affected cell. In general, these repair pathways involve endocytosis to internalize damaged membranes, exocytosis to shed damaged membranes, and membrane patching to reseal any damage using internal endolysosome-derived donor membranes (180).

Perforin-1 insertion primarily triggers membrane patching using lysosomal and endosomal donor membranes (140, 181–183). It has also been observed that, in addition to patching, perforin-1-mediated membrane destabilization promotes clathrin- and dynamin-dependent endocytosis resulting in the internalization of both perforin-1 and granzymes (183–185). These data led to the hypothesis that endolysosomal compartments (gigantosomes) rather than the plasma membrane are the primary site of perforin-1 pore assembly and granzyme entry into the cytosol. Later studies, however, did not support this model. Firstly, it remains controversial whether the luminal pH and Ca^2+^ concentration in gigantosomes are permissive for assembly of perforin-1 pores (140, 186). Secondly, the relatively slow kinetics of granzyme endocytosis and release (~ 15 min) are inconsistent with the rapid (~ 2 min) induction of Bid cleavage reported in cells exposed to granzyme B and sublytic amounts of perforin-1 (140). Hence, Lopez et al. proposed that, while membrane repair pathways do indeed negatively regulate perforin-1 activity at the plasma membrane, they allow the formation of transient pores that persist for 20–80 s providing sufficient permeability to deliver granzymes into the cytosol of the target cell and to initiate apoptosis (140). Future work involving high-resolution electron tomography might be necessary to resolve the controversy surrounding the primary location of functional perforin-1 pores, but what remains clear is that membrane repair pathways are important to prevent uncontrolled perforin-1 mediated lysis of the target cells.

Ca^2+^ influx and membrane repair have also been reported upon membrane insertion of gasdermins (187). Gasdermin pores trigger recruitment of endosomal sorting complexes required for transport (ESCRTs) which mediate repair of damaged membranes through exocytosis (188). ESCRT-mediated membrane repair negatively regulates GSDMD-induced pyroptotic death as well as the release of IL-1β and IL-18 from infected cells (187). Considering ESCRTs are also recruited to membranes exposed to CDCs such as streptolysin O and listeriolysin O (188), it appears likely that they may contribute to the removal of perforin-1 pores as well. Interestingly, ESCRTs are also involved in repair of small perforations in endolysosomes to facilitate recovery of damaged intracellular membranes (189). This pathway might provide a safety mechanism against possible accidental damage of PFP storage compartments and an additional layer of protection for PFP-producing cells.

## Concluding Remarks

It is striking how both innate and adaptive immune systems employ PFPs as their key effectors. In this review, we aimed to provide an overview of the pathways and immune system components involved in controlling the activity of these membrane-disrupting molecules. However, it is important to recognize that PFP biology is tightly linked to fundamental processes that go well beyond what we discussed here including positive and negative T cell selection, antibody affinity maturation as well as signaling pathways associated with different types of cell death, all of which ultimately contribute to the regulation of PFP activity.

Despite the century of research since the discovery of MAC, many questions regarding PFPs remain unanswered. Is extrahepatic production of MAC components physiologically relevant during infections? What are the precise conformational changes that govern the initial membrane insertion of MAC and the final pore closure? Is mechanopotentiation involved in protecting degranulating CTLs and NKs cells from perforin-1 activity *in vivo*? How is translation of perforin-1 and other NK effectors suppressed in resting cells?

The recent discovery of perforin-2 and gasdermins has also opened new avenues to explore. What are the physiological functions of all gasdermins? Are they able to assemble on endolysosomal membranes and if so, what are the consequences of the potential damage? Is perforin-2 released from the phagosomal membrane to form pores on intravacuolar pathogens and if so, by which proteases? Does perforin-2 damage phagosomes or does it assemble exclusively on bacterial membranes?

The latest advances in the PFP field have uncovered an unexpected link between perforin-1-mediated granzyme delivery and gasdermin activation, and future work is likely to reveal other examples of such cooperativity. We now also only begin to appreciate the different mechanisms involved in the sensing and repair of damaged membranes and how they can affect the consequences of pore formation. Finally, many of the regulatory pathways discussed in this review can be disrupted by pathogens, and the full picture of the mechanisms involved in evasion of PFP-mediated immunity is yet to emerge.

There is no doubt that the PFP field continues to rapidly expand. Following the recent advances in cryo-electron microscopy, the structures of MAC, perforin-1/2, and gasdermin pores are now available shedding some light on the conformational changes involved in pore assembly. Whole exome sequencing data from immunodeficient patients is helping to uncover novel disease-associated variants both in PFPs themselves and in their regulators. Finally, CRISPR-Cas9 technology is facilitating the generation of cell-type specific knockouts to address the contribution of candidate proteins in PFP biology using primary cells and animal models of disease. Novel scalable assays to study PFPs *in vitro*, identification of co-regulators through genetic screens in the relevant primary cell types, and structural insights into pre-pore and pore intermediates should provide us with a more complete picture of the mechanisms involved in the regulation of these powerful effectors and facilitate development of targeted immunomodulatory therapeutics.

## Author Contributions

All authors listed have made a substantial, direct, and intellectual contribution to the work and approved it for publication.

## Funding

We would like to thank Dr. Greg Slodkowicz for comments on the manuscript. The authors were supported by the MRC grant MC_UP_1201/26. PAK was also supported by a Boehringer Ingelheim Fonds PhD Fellowship and the Cambridge Trust.

## Conflict of Interest

The authors declare that the research was conducted in the absence of any commercial or financial relationships that could be construed as a potential conflict of interest.
